# Comparison of VTE prophylaxis agents on hemoglobin levels after total knee arthroplasty: a hospital information system-based observational study

**DOI:** 10.1186/s13018-025-06004-7

**Published:** 2025-06-16

**Authors:** Liang Yuan, Bin Sun, Xing Xin, Xinguang Liu, Carmela Pizzigallo, Stefano Gumina, Xiaohua Wang, Bin Yang

**Affiliations:** 1 Department of Orthopaedics, Peking University International Hospital, Life Park Road No.1, Life Science Park of Zhongguancun, Changping District, Beijing, 102206 China; 2 Department of Anatomy, Histology, Legal Medicine, and Orthopaedics, Sapienza University of Rome, Rome, Italy

**Keywords:** Venous thromboembolis, Pharmacologic prophylaxis, Total knee arthroplasy, Hemoglobin decline

## Abstract

**Background:**

While venous thromboembolism (VTE) prophylaxis is crucial following major orthopaedic surgeries including total knee arthroplasty (TKA), the impact of different prophylactic agents on postoperative hemoglobin (Hb) levels remains inadequately studied. The aim of this study was to compare the effects of aspirin, rivaroxaban, and low-molecular-weight heparin (LMWH) on early postoperative Hb changes following TKA.

**Methods:**

In this single-center retrospective cohort study, 655 primary TKAs were finally included using data from the hospital information system. Patients received either aspirin, rivaroxaban, or LMWH for VTE prophylaxis. The primary outcome was the magnitude of Hb reduction, calculated as the difference between the Hb level on the first postoperative day and the minimum postoperative Hb level before discharge. The secondary outcome was the trajectory of postoperative Hb changes within the first week.

**Results:**

Postoperative Hb levels clearly declined within the first week, with a mean of 13.9 g/L (*SD*, 8.5) from postoperative day 1 in the entire cohort. In the fully adjusted linear regression model, both rivaroxaban (*β* = 1.5, [95%*CI*, 0.0 to 3.0]) and low-molecular-weight heparin (LMWH) (*β* = 3.3, [95%*CI*, 1.5 to 5.2]) were associated with a greater reduction in Hb compared to aspirin. Regarding the trajectory of postoperative Hb changes, the generalized additive mixed model revealed no statistically significant difference between rivaroxaban and aspirin (*β* = -0.2, [95%*CI*, -0.6 to 0.2]). LMWH was associated with a greater daily reduction in Hb levels relative to aspirin, averaging 0.8 g/L per day (*β* = -0.8, [95%*CI*, -1.3 to -0.3]). Despite these observed differences, the effect sizes were small, suggesting a lack of clinical significance.

**Conclusions:**

Hemoglobin levels declined significantly following TKA within the first week after surgery. However, no clinically meaningful distinction is discernible between the three most frequently used pharmacological agents, aspirin, rivaroxaban and LMWH.

**Level of evidence:**

III, A retrospective cohort study.

**Supplementary Information:**

The online version contains supplementary material available at 10.1186/s13018-025-06004-7.

## Background

Venous thromboembolism (VTE) is a significant complication in orthopaedic patients [1, 2], particularly following major surgeries such as hip and knee arthroplasties [3–6]. Prevention of VTE has become a critical component of perioperative management in major orthopedic surgeries. A number of studies have demonstrated that the use of pharmacological agents can effectively reduce the incidence of VTE [7, 8]. Currently, the main drugs employed in this context include low-molecular-weight heparin (LMWH), the new direct oral anticoagulants such as rivaroxaban, and aspirin. In terms of efficacy in preventing VTE, these drugs have been supported by different studies [9–15]. However, the optimal choice of therapeutic agents remains controversial, particularly with regard to the haemorrhagic risk of these drugs [16, 17].

In the process of VTE prevention, clinicians typically prioritize the risk of bleeding, which has become a crucial aspect of evaluating the safety of these drugs [18]. There has been an assumption that anticoagulants (e.g., LMWH, the new oral anticoagulants) will result in a greater incidence of bleeding complications and a more pronounced reduction in hemoglobin (Hb) levels than aspirin [19]. Nevertheless, there is no consistent evidence to support this view [16]. Furthermore, current clinical studies are mainly focused on the incidence of bleeding events, blood transfusion and the assessment of occult blood loss [15, 17, 20]. There has been comparatively little attention paid to the more intuitive measure of the decrease in Hb level after surgery. There is a paucity of studies that have employed postoperative Hb decline as an outcome measure to assess the safety of VTE prophylaxis. It is our contention that the decrease in Hb level, though not a direct proxy for blood loss, can serve as an intuitive reflection of the anemia status and severity of the patient. It can facilitate a straightforward and transparent approach for physicians to assess the condition of patients and adjust the treatment in a timely manner, thereby promoting the rapid recovery after surgery.

Knee arthroplasty, as one of the major orthopedic surgeries, has gained increasing attention, with a rising trend in surgical volume [21–25]. Consequently, issues related to the prevention of VTE after knee arthroplasty have also become a significant focus. Accordingly, we conducted a retrospective observational study based on data from the hospital information system (HIS) with the aim of investigating the impact of VTE prophylaxis medications on the reduction of Hb levels following primary total knee arthroplasty (TKA) at the early stage of postoperative period.

## Methods

### Study design and data source

A retrospective cohort study was conducted utilizing data from the HIS database. Data retrieval was performed by specialized information technology staff. The International Classification of Diseases, 10th Revision (ICD-10) and the International Classification of Diseases, 9th Revision, Clinical Modification, Volume 3 (ICD-9-CM3) codes were employed to identify the records of patients undergoing primary TKA at the single-center facility. All patient information was anonymized prior to exportation from the HIS. The study protocol was approved by the institutional research ethics board (No. 2023-KY-0044-01). A waiver of written informed consent was granted due to the retrospective study design and the deidentified nature of the data. All procedures were conducted in accordance with the Declaration of Helsinki. The writing of this article also referenced the STROBE guideline (https://www.strobe-statement.org/) and the STROBE checklist was used.

### Study population

**Fig. 1:**
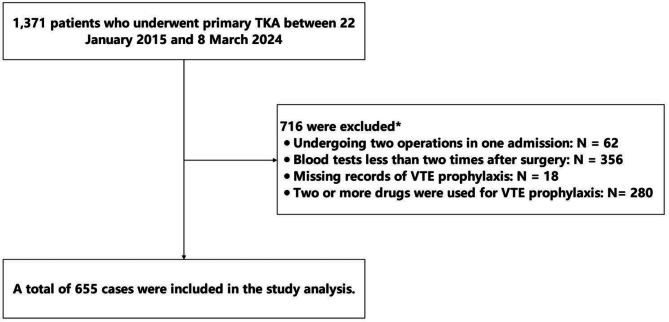
Study population. The asterisk denotes that the subjects met the combined conditions. Abbreviations: TKA = Total knee arthroplasty; VTE = Venous Thromboembolism

The present study involved a comprehensive analysis of medical records for 1,371 patients who underwent primary TKA between 22 January 2015 and 8 March 2024. Cases were excluded if they met the following criteria: (1) undergoing two operations in one admission, (2) blood tests less than two times after surgery, (3) missing records of VTE prophylaxis, (4) two or more drugs were used for VTE prophylaxis, as illustrated in Fig. 1. A total of 655 participants were finally included, using different VTE prophylaxis medications, including aspirin (*n* = 275), rivaroxaban (*n* = 249 ), and LMWH (*n* = 131). Baseline Characteristics of participants were shown in Table 1.

**Table 1:** Baseline characteristics of participants

Characteristics	Total	VTE prophylaxis medications			*P*-value
		Aspirin	Rivaroxaban	LMWH	
No. of participants	655	275	249	131	
Age (years, mean ± *SD*)	68.1 ± 6.7	68.1 ± 7.0	67.6 ± 6.5	69.0 ± 6.4	0.142
Hb decline (g/L, mean ± *SD*)	13.9 ± 8.5	12.8 ± 8.3	14.1 ± 8.8	15.9 ± 8.0	0.003
BMI	27.0 ± 3.6	26.8 ± 3.6	27.1 ± 3.7	27.4 ± 3.5	0.404
LOS (days, mean ± *SD*)	14.0 ± 5.8	15.3 ± 6.2	13.6 ± 5.5	12.4 ± 4.9	< 0.001
Postoperative LOS (days, mean ± *SD*)	10.7 ± 4.6	11.4 ± 4.9	10.4 ± 4.5	9.7 ± 4.3	0.002
Preoperative Hb level (g/L, mean ± *SD*)	129.6 ± 14.1	130.8 ± 14.1	130.1 ± 12.7	125.9 ± 16.0	0.002
Sex, n (%)					0.056
Women	535 (81.7%)	214 (77.8%)	214 (85.9%)	107 (81.7%)	
Men	120 (18.3%)	61 (22.2%)	35 (14.1%)	24 (18.3%)	
Ethnicity, n (%)					0.204
Han nationality	627 (95.7%)	267 (97.1%)	234 (94.0%)	126 (96.2%)	
Others	28 (4.3%)	8 (2.9%)	15 (6.0%)	5 (3.8%)	
KOA, n (%)					0.763
No	22 (3.4%)	8 (2.9%)	10 (4.0%)	4 (3.1%)	
Yes	633 (96.6%)	267 (97.1%)	239 (96.0%)	127 (96.9%)	
CHD, n (%)					0.337
No	593 (90.5%)	248 (90.2%)	230 (92.4%)	115 (87.8%)	
Yes	62 (9.5%)	27 (9.8%)	19 (7.6%)	16 (12.2%)	
Hypertension, n (%)					0.858
No	268 (40.9%)	115 (41.8%)	102 (41.0%)	51 (38.9%)	
Yes	387 (59.1%)	160 (58.2%)	147 (59.0%)	80 (61.1%)	
DM, n (%)					0.22
No	477 (72.8%)	201 (73.1%)	188 (75.5%)	88 (67.2%)	
Yes	178 (27.2%)	74 (26.9%)	61 (24.5%)	43 (32.8%)	
Use of PSI, n (%)					< 0.001
No	473 (72.2%)	229 (83.3%)	178 (71.5%)	66 (50.4%)	
Yes	182 (27.8%)	46 (16.7%)	71 (28.5%)	65 (49.6%)	
Preoperative anemia, n (%)					0.001
No	594 (90.7%)	253 (92.0%)	233 (93.6%)	108 (82.4%)	
Yes	61 (9.3%)	22 (8.0%)	16 (6.4%)	23 (17.6%)	
ASA grade, n (%)					0.696
ASA I	11 (1.8%)	6 (2.2%)	3 (1.3%)	2 (1.6%)	
ASA II	514 (82.0%)	224 (83.6%)	184 (80.0%)	106 (82.2%)	
ASA III	101 (16.1%)	38 (14.2%)	42 (18.3%)	21 (16.3%)	
ASA IV	1 (0.2%)	0 (0.0%)	1 (0.4%)	0 (0.0%)	
Tranexamic acid, n (%)					< 0.001
No	243 (37.1%)	77 (28.0%)	96 (38.6%)	70 (53.4%)	
Yes	412 (62.9%)	198 (72.0%)	153 (61.4%)	61 (46.6%)	
HOCA, n (%)					0.858
No	571 (87.2%)	242 (88.0%)	216 (86.7%)	113 (86.3%)	
Yes	84 (12.8%)	33 (12.0%)	33 (13.3%)	18 (13.7%)	
Blood transfusion, n (%)					0.852
No	616 (94.0%)	260 (94.5%)	234 (94.0%)	122 (93.1%)	
Yes	39 (6.0%)	15 (5.5%)	15 (6.0%)	9 (6.9%)	
Postoperative DVT, n (%)					0.757
No	638 (97.4%)	267 (97.1%)	244 (98.0%)	127 (96.9%)	
Yes	17 (2.6%)	8 (2.9%)	5 (2.0%)	4 (3.1%)	

Abbreviations: BMI = Body Mass Index; LOS = Length of Stay; VTE = Venous Thromboembolism; LMWH = low-molecular-weight heparin; Hb = Hemoglobin; KOA = Knee Osteoarthritis; CHD = Coronary Heart Disease; DM = Diabetes Mellitus; PSI = Patient specific instrumentation; ASA = American Society of Anesthesiologists; HOCA = history of other conditions that may require anticoagulation or antiplatelet therapy; DVT = Deep vein thrombosis

### Variables and measurements

Routine blood tests were conducted prior to surgery and subsequently repeated at intermittent intervals from the first postoperative day until the day of discharge. In our hospital, patients undergoing TKA typically preferred to remain hospitalized until it was more convenient for them to move, so they generally would like to stay for 7 to 14 days or more after surgery, which provided a favorable condition for us to dynamically monitor changes in Hb levels. The distributions of postoperative blood test times across the cohort and the days between postoperative blood tests were shown in eTable 1 and eTable 2, respectively, in the Supplementary Materials. The blood tests were conducted by an independent clinical laboratory within the hospital. The laboratory personnel were blinded to the clinical information pertaining to the patients. The primary outcome of this study was the reduction in Hb levels in the early postoperative period. It was defined as the Hb value on the first day after surgery minus the minimum value of postoperative Hb levels before discharge. It was not set as the preoperative Hb minus the postoperative minimum to eliminate the influence of overt blood loss during surgery. The secondary outcome measure was the trend of postoperative changes in Hb levels. The exposure factor in this study was the option of prophylactic medications for VTE as detailed in the treatment protocol.

Demographic data and information on potential factors related to Hb levels were extracted from medical records. These factors were included in the analysis as covariates.

### Treatment protocol

In the absence of contraindications, VTE prophylaxis was administered from the first postoperative day onwards. The drug was selected by the chief surgeon from one of three options, aspirin, rivaroxaban, or LMWH. The choice was based on the surgeon’s expertise. Medications were typically started on the first postoperative day. During hospitalization, patients were given either aspirin 100 mg once daily, rivaroxaban 10 mg once daily or LMWH, dosed according to body weight, 4000 IU once daily for those weighing less than 60 kg and 6000 IU once daily for those weighing more than 60 kg, in accordance with the guidelines for VTE prophylaxis [26, 27]. Other treatments included infection prophylaxis, analgesia, and detumescence therapy. Apart from differences in VTE prophylaxis, postoperative management was basically homogeneous.

All surgical procedures, despite being performed by multiple surgeons, were largely consistent, employing a medial parapatellar approach through a midline incision. A notable variation was the application of patient specific instrumentation (PSI) technology in certain cases, which obviated the need for femoral medullary cavity reaming, hence, it was considered as a covariate in the analysis. Some cases involved the intraoperative use of tranexamic acid, which was also included as a covariate in the analysis. As previously mentioned, postoperative blood tests were routinely conducted at intervals to monitor trends in Hb levels. During the monitoring of Hb levels, a transfusion may be indicated if the Hb was excessively low. For individual patients, the decision to transfuse must consider both the Hb results and whether the patient exhibits symptoms of anemia, as well as the availability of blood supplies. Consequently, transfusion criteria were not uniform. However, the timing of transfusion generally occurred after the lowest detected Hb value. Therefore, the outcome measure of Hb decline (as defined previously) in this study would not be influenced by the transfusion intervention. Systematic DVT screening for deep vein thrombosis (DVT) following TKA was not performed; typically, venous ultrasonography would be conducted only when there was a high clinical suspicion of DVT to confirm the diagnosis.

### Data analyses

All analyses were performed using EmpowerStats software (www.empowerstats.com, X & Y solutions, Inc., Boston, MA, USA) and R (https://www.r-project.org/). Descriptive analyses were run for demographics and clinical characteristics. Categorical variables were presented as count (%) and continuous measurements were presented as mean ± SD. A two-sided *P-*value < 0.05 was considered statistically significant. Baseline characteristics of all patients in the different VTE prophylaxis groups were assessed using one-way ANOVA or Kruskal-Wallis rank-sum test for continuous data and chi-squared test for categorical variables (Table 1). A preliminary examination of the relationship between covariates and the outcome variable (Hb decline, g/L) was conducted using univariate analysis with no ajusted variables (Table 2).

**Table 2:** Univariate analysis for Hb decline (g/L)

Covariates	Statistics	β (95%CI)	*P*-value
Age, year	68.09 ± 6.69	0.1 (-0.0, 0.2)	0.144
BMI	27.03 ± 3.61	-0.1 (-0.3, 0.1)	0.239
Sex			
Women	535 (81.68%)	0	
Men	120 (18.32%)	3.7 (2.1, 5.4)	<0.001
Ethnicity			
Han nationality	627 (95.73%)	0	
Others	28 (4.27%)	-1.1 (-4.4, 2.1)	0.489
KOA			
No	22 (3.36%)	0	
Yes	633 (96.64%)	0.0 (-3.6, 3.6)	0.999
CHD			
No	593 (90.53%)	0	
Yes	62 (9.47%)	2.7 (0.5, 4.9)	0.017
Hypertension			
No	268 (40.92%)	0	
Yes	387 (59.08%)	0.7 (-0.6, 2.0)	0.317
DM			
No	477 (72.82%)	0	
Yes	178 (27.18%)	0.2 (-1.2, 1.7)	0.743
PSI			
No	473 (72.21%)	0	
Yes	182 (27.79%)	1.2 (-0.3, 2.6)	0.114
Preoperative anemia			
No	594 (90.69%)	0	
Yes	61 (9.31%)	-3.0 (-5.2, -0.8)	0.008
ASA grade			
ASA I	11 (1.75%)	0	
ASA II	514 (81.98%)	-1.7 (-6.7, 3.2)	0.493
ASA III	101 (16.11%)	-0.2 (-5.4, 5.0) 0.939	0.939
ASA IV	1 (0.16%)	-10.3 (-27.3, 6.8)	0.237
VTE prophylaxis medications			
Aspirin	275 (41.98%)	0	
Rivaroxaban	249 (38.02%)	1.3 (-0.2, 2.7)	0.089
LMWH	131 (20.00%)	3.1 (1.3, 4.8)	< 0.001
Tranexamic acid			
No	243 (37.10%)	0	
Yes	412 (62.90%)	-1.0 (-2.3, 0.4)	0.158
HOCA			
No	571 (87.2%)	0	
Yes	84 (12.8%)	0.0 (-1.9, 2.0)	0.984

Abbreviations: *OR* = Odds Ratio; 95%*CI* = 95% Confidence Interval; BMI = Body Mass Index; VTE = Venous Thromboembolism; LMWH = Low-molecular-weight Heparin; Hb = Hemoglobin; KOA = Knee Osteoarthritis; CHD = Coronary Heart Disease; DM = Diabetes Mellitus; PSI = Patient specific instrumentation; ASA = American Society of Anesthesiologists; HOCA = history of other conditions that may require anticoagulation or antiplatelet therapy

Linear regression analysis was used for multivariable analysis (Table 3). The associations between VTE prophylaxis medications and Hb decline were analyzed. Crude model adjusted for no covariates. Model I adjusted for age, sex, ethnicity. Model II adjusted for age, sex, ethnicity, BMI, diagnosis of KOA, history of hypertension, history of diabetes, history of coronary heart disease, use of PSI, ASA grade, preoperative anemia, use of tranexamic acid during surgery, and history of other conditions that may require anticoagulation or antiplatelet therapy (HOCA), such as old cerebral infarction, atrial fibrillation and arterial disease other than coronary heart disease.

**Table 3:** Association between VTE prophylaxis medications and Hb decline (g/L) in different models

Variable	Non-adjusted (β, 95%CI, *P*)	Model I (β, 95%CI, *P*)	Model II (β, 95%CI, *P*)
VTE prophylaxis medications			
Aspirin	0 (reference)	0 (reference)	0 (reference)
Rivaroxaban	1.3 (-0.2, 2.7) 0.089	1.6 (0.2, 3.1) 0.027	1.5 (0.0, 3.0) 0.045
LMWH	3.1 (1.3, 4.8) < 0.001	3.2 (1.4, 4.9) < 0.001	3.3 (1.5, 5.2) < 0.001

Abbreviations: 95%CI = 95% Confidence Interval; VTE = Venous Thromboembolism; Hb = Hemoglobin;LMWH = Low-molecular-weight Heparin; BMI = Body Mass Index; KOA = Knee Osteoarthritis; PSI = Patient specific instrumentation; ASA = American Society of Anesthesiologists; HOCA = history of other conditions that may require anticoagulation or antiplatelet therapy

Model I: adjusted for age, sex, ethnicity

Model II: adjusted for age, sex, ethnicity, BMI, diagnosis of KOA, history of hypertension, history of diabetes, history of coronary heart disease, use of PSI, ASA grade, preoperative anemia, use of tranexamic acid during surgery, HOCA

**Fig. 2:**
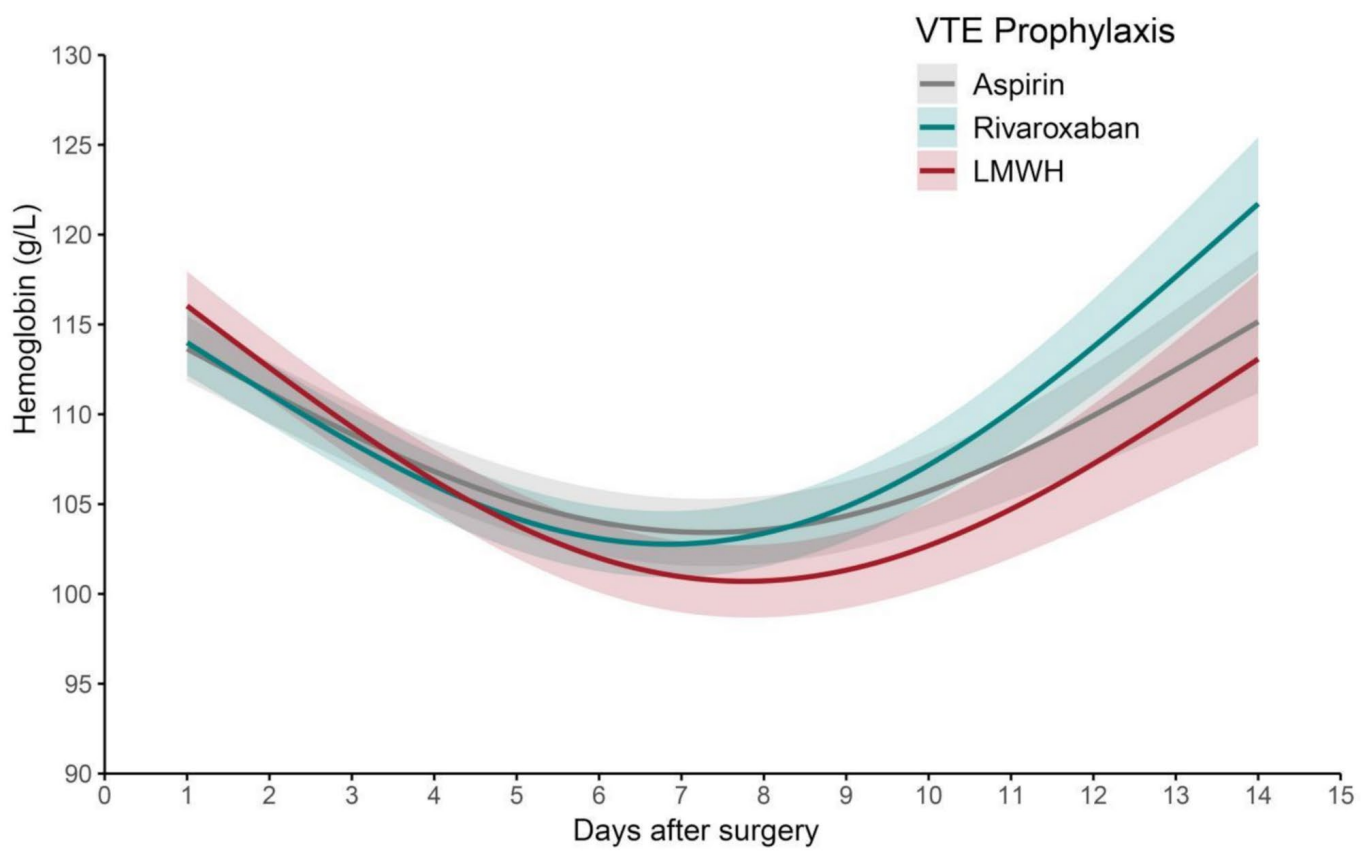
Association between changes in Hb levels (g/L) and VTE prophylaxis medications. A non-linear association between changes in Hb levels and VTE prophylaxis medications was observed in a generalized additive model (GAM). Smooth curve fitting graph illustrated Hb levels based on the days after surgery. The grey line represented Aspirin. The green line represented Rivaroxaban. The red line represented LMWH. All adjusted for age, sex, ethnicity, BMI, diagnosis of KOA, history of hypertension, history of diabetes, history of coronary heart disease, use of PSI, ASA grade, preoperative anemia, use of tranexamic acid during surgery, HOCA, and blood transfusion. Abbreviations: Hb = Hemoglobin; VTE = Venous Thromboembolism; LMWH = Low-molecular-weight Heparin; BMI = Body Mass Index; KOA = Knee Osteoarthritis; PSI = Patient specific instrumentation; ASA = American Society of Anesthesiologists; HOCA = history of other conditions that may require anticoagulation or antiplatelet therapy

A generalized additive model (GAM) with smooth curve fitting was used to investigate postoperative changes of Hb level over time between groups of different VTE prophylaxis medications (Fig. 2). Subsequently, a generalized additive mixed model (GAMM) was employed to analyze the relationship between VTE prophylaxis and early (1–7 days) changes in postoperative Hb level (Table 4). Both the GAM and GAMM are particularly advantageous for analyzing repeated measures, particularly in situations characterized by irregular intervals between measurements, potential missing data, and moderate sample sizes [28–30]. This methodological approach enhances the robustness and validity of the findings regarding the effects of VTE prophylaxis on postoperative Hb levels.

**Table 4:** Relationship between VTE prophylaxis medications and changes in Hb levels (g/L) during the early postoperative period (1–7 days) in 655 patients derived from a generalized additive mixed model (GAMM)

Outcome	Model I		Model II	
	β(95%CI)	*P*-value	β(95%CI)	*P*-value
(Intercept)	121.6 (112.7, 130.5)	< 0.001	105.2 (92.7, 117.7)	< 0.001
Day	-2.1 (-2.4, -1.8)	< 0.001	-2.0 (-2.3, -1.7)	< 0.001
Factor(VTE prophylaxis medications)1	-0.7 (-2.8, 1.4)	0.489	-0.7 (-2.7, 1.3)	0.497
Factor(VTE prophylaxis medications)2	1.7 (-0.8, 4.2)	0.188	3.1 (0.6, 5.5)	0.016
Day × Factor(VTE prophylaxis medications)1	-0.2 (-0.6, 0.2)	0.430	-0.2 (-0.6, 0.2)	0.338
Day × Factor(VTE prophylaxis medications)2	-0.8 (-1.2, -0.3)	0.002	-0.8 (-1.3, -0.3)	< 0.001

Abbreviations: 95%CI = 95% Confidence Interval; VTE = Venous Thromboembolism; Hb = Hemoglobin;LMWH = Low-molecular-weight Heparin; BMI = Body Mass Index; KOA = Knee Osteoarthritis; PSI = Patient specific instrumentation; ASA = American Society of Anesthesiologists; HOCA = history of other conditions that may require anticoagulation or antiplatelet therapy

***Intercept***, the mean of Hb level at day = 0 and VTE prophylaxis medications = 0 (aspirin), ***Day***, the mean of the increasing of Hb level at VTE prophylaxis medications = 0 (aspirin) over time (daily), ***Factor(VTE prophylaxis medications)1***, the difference of Hb level at day = 0 between the group of VTE prophylaxis medications = 1 (rivaroxaban) and the group of VTE prophylaxis medications = 0 (aspirin), ***Factor(VTE prophylaxis medications)2***, the difference of Hb level at day = 0 between the group of VTE prophylaxis medications = 2 (LMWH) and the group of VTE prophylaxis medications = 0 (aspirin), ***Day × Factor(VTE prophylaxis medications)1***, the average increasing in Hb level daily under the condition of the group of VTE prophylaxis medications = 1 (rivaroxaban) compared with the group of VTE prophylaxis medications = 0 (aspirin), ***Day × Factor(VTE prophylaxis medications)2***, the average increasing in Hb level daily under the condition of the group of VTE prophylaxis medications = 2 (LMWH) compared with the group of VTE prophylaxis medications = 0 (aspirin).

Model I: adjusted for age, sex, ethnicity

Model II: adjusted for age, sex, ethnicity, BMI, diagnosis of KOA, history of hypertension, history of diabetes, history of coronary heart disease, use of PSI, ASA grade, preoperative anemia, use of tranexamic acid during surgery, HOCA, and blood transfusion

Two variables, BMI and ASA grade, had missing data. As sensitivity analyses, and to maximize statistical power and minimize potential bias that may arise from the exclusion of missing data, we employed a multiple imputation method based on five imputations and the chained equation approach implemented in the R MI program to handle missing data [31–33]. We repeated the linear regression analysis using the full dataset for comparison. Further details of the statistical analyses are provided in eTable 3 in the Supplementary Materials.

Fully adjusted model for linear regression, GAM, and GAMM all adjusted for the same covariates, age, sex, ethnicity, BMI, diagnosis of KOA, history of hypertension, history of diabetes, history of coronary heart disease, use of PSI, ASA grade, preoperative anemia, use of tranexamic acid during surgery and history of other conditions that may require anticoagulation or antiplatelet therapy (HOCA), such as old cerebral infarction, atrial fibrillation and arterial disease other than coronary heart disease. Variables considered to be confounders based on existing literature [34–36] and clinical judgement were also included.

## Results

### Characteristics of study population

In this study, a total of 655 participants were finally included, using different VTE prophylaxis medications, including aspirin (275 participants), rivaroxaban (249 participants), and LMWH (131 participants). The mean age was 68.1 years, with no significant differences among the groups in terms of age, BMI, and gender distribution (*p*-values of 0.142, 0.404, and 0.056, respectively). However, significant differences were observed in preoperative Hb level (*p* = 0.002), preoperative anemia (*p* = 0.001), Hb decline (*p* = 0.003), use of PSI (*p* < 0.001), and the use of tranexamic acid (*p* < 0.001) among the different medication groups. These findings provide important insights into the baseline characteristics of participants across various VTE prophylaxis treatments. (Table 1)

### Univariate analysis

The univariate analysis for Hb decline revealed several significant associations. Sex was found to be a strong predictor, with men having an increased risk (*β* = 3.7, *P* < 0.001) compared to women. The history of coronary heart disease (CHD) also demonstrated a significant effect on Hb decline (*β* = 2.7, *P* = 0.017). Notably, preoperative anemia was associated with a considerable decrease in Hb levels (*β* = -3.0, *P* = 0.008). Furthermore, LMWH showed a significant positive association with Hb decline (*β* = 3.1, *P* = 0.001). Other variables, such as age, BMI, ethnicity, history of hypertension, history of diabetes mellitus, use of PSI, ASA grade, the use of tranexamic acid, and HOCA, did not exhibit significant associations with Hb decline (*P*-values ranging from 0.144 to 0.984). (Table 2)

### The relationship between VTE prophylaxis medications and hb decline across crude and adjusted models

The analysis of the relationship between VTE prophylaxis medications and Hb decline across different models showed that rivaroxaban and LMWH were associated with increased Hb decline compared to aspirin. In the non-adjusted model, rivaroxaban had a *β* of 1.3 (95% *CI*, -0.2-2.7, *P* = 0.089), which became significant in the adjusted models, Model I (*β* = 1.6, 95% *CI*, 0.2–3.1, *P* = 0.027) and Model II (*β* = 1.5, 95% *CI*,0.0–3.0, *P* = 0.045). LMWH consistently showed a strong association with Hb decline across all models, with *β* values of 3.1, 3.2, and 3.3 (95% *CIs* ranging from1.3 to 5.2, *P* < 0.001). (Table 3)

### Changes in hb levels (g/L) during the early postoperative period (1–14 days)

In Fig. 2, we can observe the changes in Hb levels (g/L) over a 14-day period following surgery for patients receiving different VTE prophylaxis medications, Aspirin, Rivaroxaban, and LMWH. Hb levels initially decreased, reaching a nadir around day 7, after which they gradually increased. The trajectory of Hb levels varied little among the medication groups.

### Relationship between VTE prophylaxis medications and changes in Hb levels (g/L) during the early postoperative period (1–7 days)

As shown in Table 4, we determined the relationship between VTE prophylaxis medications and changes in Hb levels (g/L) during the early postoperative period (1–7 days). Regarding the trajectory of postoperative Hb changes, the generalized additive mixed model (GAMM) revealed no statistically significant difference between rivaroxaban and aspirin (*β* = -0.2, 95%*CI*, -0.6 to 0.2, *P* = 0.338). In comparison, LMWH was associated with a greater daily reduction in Hb levels relative to aspirin, averaging 0.8 g/L (0.08 g/dL) per day (*β* = -0.8, 95%*CI*, -1.3 to -0.3, *P* < 0.001). We concluded that there were no clinically significant differences in Hb levels between the groups of VTE prophylaxis medications.

## Discussion

In the context of VTE prophylaxis in orthopaedics, this study elucidated the trends in Hb levels in patients undergoing primary TKA through an analysis of repeated measures data, with a particular emphasis on comparing the impact of different pharmacological interventions for VTE prophylaxis on early postoperative Hb decline. The findings provided a foundational basis for pharmacological strategies during the perioperative management of TKA. Data from this study indicated a significant decline in Hb levels during the first week post-operation, with an average decrease of 13.9 g/L, followed by a gradual recovery, a trend consistent with our clinical observations. Specifically, when examining the three commonly used VTE prophylactic medications, statistical differences were observed among the groups, however, the effect sizes were very small, lacking significant clinical relevance. Although the multivariate regression analysis (Table 3) indicated significant statistical differences between the different groups (*P* < 0.05), this result merely confirms that our statistical power is adequate. Nonetheless, the small effect sizes (*β* values were 1.5 for rivaroxaban vs. aspirin and 3.3 for LMWH vs. aspirin) suggested that, based on clinical judgement, differences in Hb levels of 1.5 g/L and 3.3 g/L had no practical clinical significance. Similar situation was also seen in the relationship between VTE prophylaxis medications and changes in Hb levels (g/L) during the early postoperative period (1–7 days) derived from the GAMM (Table 4).

The primary efficacy of pharmacological interventions for VTE prophylaxis was to reduce the tendency of blood to coagulate, which inherently raised the potential side effect of increased bleeding. Consequently, clinicians paid particular attention to bleeding-related events and indicators following the administration of these medications. Previous studies also explored this issue [15, 37]. Cheng-fong Chen and Shang-Wen Tsai et al. [37] evaluated the effects of continued aspirin mono-therapy on calculated blood loss and transfusion requirements, reporting that patients on aspirin had a calculated blood loss of 969.1 mL and a transfusion rate of 53.0%, compared to 904.0 mL and 40.2% in the non-aspirin cohort. In a meta-analysis, Ze-Nan Xia and Qing Zhou et al. [38] demonstrated that LMWH significantly reduced the incidence of DVT (*OR*, 0.57) and wound complications, albeit with an associated increase in bleeding events and total blood transfusion (*OR*, 1.57). These findings emphasize the effective role of LMWH in DVT prophylaxis while raising concerns regarding its impact on bleeding. This has helped us to understand the benefits and risks of LMWHs, but there has been a lack of comparison with other commonly used drugs. In contrast, our study primarily elucidated trends in Hb levels in the early postoperative period, culminating in an average decline of 13.9 g/L (*SD*, 8.5) within the first week, with minimal differences observed among different VTE prophylaxis medications. While both studies indicate clinical concerns regarding blood management in TKA, the previous published research highlights specific bleeding risks and transfusion metrics related to aspirin use, whereas our findings contribute to understanding the trend of Hb levels in relation to VTE prophylaxis strategies. This divergence in focus enriches the discourse on optimizing perioperative management in TKA.

Summarising previous studies, we found that outcomes mainly included bleeding, wound drainage and transfusion rates [15, 37–39]. Although these outcome measures have notable clinical relevance and provide valuable guidance in clinical practice, they also come with certain limitations that should be carefully considered. It is important to acknowledge that clinically observable bleeding events tend to occur only when the degree of coagulopathy reaches a specific threshold [40]. Occult bleeding, which is bleeding that is not visible or detectable without further investigation, is relatively common during the postoperative period [41, 42]. This indicates that bleeding events may not sufficiently reflect the differences in blood loss that may occur after VTE prophylaxis are administered. Furthermore, wound drainage is a parameter frequently used to gauge the amount of early manifest blood loss. However, several variables can influence wound drainage measurements [43]. Furthermore, based on our clinical observations, factors such as the positioning of the drainage tube, its patency, and the timing of drainage tube removal may also influence the accuracy of recorded drained volume. Another critical aspect involves the evaluation of transfusion rates, which tends to exhibit comparatively low objectivity. The reported transfusion rates after TKA vary widely in the literature, ranging from 2 to 70% in different cohorts [35, 44]. According to our observations, however, different surgeons may have a subjective understanding of transfusion indications, leading to variability in clinical practice. Some may adopt a more aggressive approach towards transfusion, while others may prefer a conservative strategy. This inconsistency can significantly impact the objectivity of transfusion data collected.

While we do not suggest that the outcome measures used in this study are inherently superior to those previously mentioned, research focusing on postoperative Hb levels as outcome may contribute to a more comprehensive and enriched chain of evidence in this research area and greatly enhance our understanding of pharmacological interventions for the prevention of VTE. We believe that Hb levels could serve as a simple yet comprehensive indicator of the postoperative hematological status of patients affected by both overt and covert blood loss. By conducting intermittent repeated measurements, observing the trends in Hb levels can enhance our understanding of the patients’ overall condition and recovery progress. Comparing the extent of early postoperative Hb decline under different medication regimens will provide us with greater confidence in our medication decision-making. This study effectively allayed concerns regarding the assumption that anticoagulants would lead to a greater reduction in Hb levels compared to aspirin.

This study also has several limitations. First, data were extracted retrospectively from the HIS database. Although this study was conducted in a single centre and there was some missing data, there was little selection bias and the results remained consistent after imputation, suggesting that the missing data did not affect the core outcomes. Second, the design of this observational study allows only associations to be inferred, not causal relationships. Third, only 655 patients were included in the analysis. However, this sample size is sufficient to draw conclusions. Fourth, while our research elucidated the relationship between various commonly used pharmacological options for VTE prophylaxis and the extent of early postoperative Hb decline, caution should be exercised regarding the generalizability of the findings. As the population in this study consisted of patients undergoing primary TKA, further studies are necessary to confirm whether similar patterns apply to those undergoing revision TKA or simultaneous bilateral TKA. Fifth, although the trend of Hb variation was analyzed using the GAM model and curve fitting, and a decreasing trend was observed within 1 week; however, due to the limited amount of data in this study, the exact time of the turning point cannot be analyzed further. Larger sample sizes and further studies are still needed to make accurate estimates. Six, as with all observational studies, despite adjustment for multiple potential confounders, residual confounding issues may not have been fully addressed.

## Conclusions

Following primary TKA, a significant decline in Hb levels was observed during the initial week. Despite this notable decrease in Hb levels, our analysis revealed that there was no clinically meaningful difference among the three most frequently prescribed pharmacological agents used for VTE prophylaxis post-surgery, aspirin, rivaroxaban, and LMWH. This finding indicates that commonly used VTE prophylactic agents, including aspirin, rivaroxaban, and LMWH, following primary TKA do not necessitate prioritization of one agent over another solely based on their impact on postoperative hemoglobin levels.

## Electronic supplementary material

Below is the link to the electronic supplementary material.

Supplementary Material 1

Supplementary Material 2

Supplementary Material 3

## Data Availability

The datasets utilized and/or analyzed in the present study are available from the corresponding author upon reasonable request.

## Abbreviations

ASA: American Society of Anesthesiologists
BMI: Body Mass Index
CHD: Coronary Heart Disease
CI: Confidence Interval
DVT: Deep Vein Thrombosis
GAM: Generalized Additive Model
GAMM: Generalized Additive Mixed Model
Hb: Hemoglobin
HIS: Hospital Information System
HOCA: History of Other Conditions that may require Anticoagulation or Antiplatelet therapy
ICD-10: International Classification of Diseases, 10th Revision
ICD-9-CM3: International Classification of Diseases, 9th Revision, Clinical Modification, Volume 3
KOA: Knee Osteoarthritis
LMWH: Low-Molecular-Weight Heparin
MI: Multiple Imputation
OR: Odds Ratio
PSI: Patient Specific Instrumentation
SD: Standard Deviation
STROBE: Strengthening the Reporting of Observational Studies in Epidemiology
TKA: Total Knee Arthroplasty
VTE: Venous Thromboembolism
