# Supplementary material for: Comparison of VTE prophylaxis agents on hemoglobin levels after total knee arthroplasty: a hospital information system-based observational study
Source: J Orthop Surg Res. 2025 Jun 16;20:589. doi: 10.1186/s13018-025-06004-7 (PMC12168285; doi:10.1186/s13018-025-06004-7)
Supplement: Supplementary file 1 — Supplementary Material 1 [file 13018_2025_6004_MOESM1_ESM.docx]

**eTable 1**. Distribution of the times for the blood tests in the cohort

| Times of blood tests | n (%) |
| --- | --- |
| 2 | 235(35.9%) |
| 3 | 245(37.4%) |
| 4 | 115(17.6%) |
| 5 | 33(5.0%) |
| 6 | 15(2.3%) |
| 7 | 8(1.2%) |
| 8 | 2(0.3%) |
| 9 | 1(0.2%) |
| 10 | 1(0.2%) |
