# Supplementary material for: Comparison of VTE prophylaxis agents on hemoglobin levels after total knee arthroplasty: a hospital information system-based observational study
Source: J Orthop Surg Res. 2025 Jun 16;20:589. doi: 10.1186/s13018-025-06004-7 (PMC12168285; doi:10.1186/s13018-025-06004-7)
Supplement: Supplementary file 2 — Supplementary Material 2 [file 13018_2025_6004_MOESM2_ESM.docx]

**eTable 2**. Distribution of days between blood tests

| Days from the first blood test after surgery | n (%) |
| --- | --- |
| 0 | 655 (32.6%) |
| 1 | 57 (2.8%) |
| 2 | 314 (15.6%) |
| 3 | 175 (8.7%) |
| 4 | 269 (13.4%) |
| 5 | 141 (7.0%) |
| 6 | 135 (6.7%) |
| 7 | 47 (2.3%) |
| 8 | 46 (2.3%) |
| 9 | 45 (2.2%) |
| 10 | 29 (1.4%) |
| 11 | 30 (1.5%) |
| 12 | 35 (1.7%) |
| 13 | 14 (0.7%) |
| 14 | 2 (0.1%) |
| 15 | 7 (0.3%) |
| 16 | 5 (0.2%) |
| 17 | 1 (0.0%) |
| 18 | 1 (0.0%) |
| 19 | 2 (0.1%) |
| 21 | 1 (0.0%) |

Day 0 was the first day after surgery, Day 1 was the second day after surgery, and so on.
