# Supplementary material for: Comparison of VTE prophylaxis agents on hemoglobin levels after total knee arthroplasty: a hospital information system-based observational study
Source: J Orthop Surg Res. 2025 Jun 16;20:589. doi: 10.1186/s13018-025-06004-7 (PMC12168285; doi:10.1186/s13018-025-06004-7)
Supplement: Supplementary file 3 — Supplementary Material 3 [file 13018_2025_6004_MOESM3_ESM.docx]

**eTable 3.** Relationship between VTE prophylaxis medications and Hb decline (g/L) in different models using multiple imputation to account for missing data

| **Variable** | **Non-adjusted**  **(*β*, 95%*CI*, *P*)** | **Adjust I**  **(*β*, 95%CI, *P*)** | **Adjust II**  **(*β*, 95%*CI*, *P*)** |
| --- | --- | --- | --- |
| VTE prophylaxis medications |  |  |  |
| Aspirin | 0 | 0 | 0 |
| Rivaroxaban | 1.3 (-0.2, 2.8) 0.079 | 1.6 (0.1, 3.1) 0.031 | 1.6 (0.1, 3.0) 0.034 |
| LMWH | 3.1 (1.4, 4.8) 0.001 | 3.2 (1.5, 4.9) <0.001 | 3.4 (1.5, 5.3) <0.001 |

**Abbreviations:** 95%CI = 95% Confidence Interval; VTE = Venous Thromboembolism; LMWH = Low-molecular-weight Heparin; BMI = Body Mass Index; KOA = Knee Osteoarthritis; PSI = Patient specific instrumentation; ASA = American Society of Anesthesiologists; HOCA = history of other conditions that may require anticoagulation or antiplatelet therapy

**Model I:** adjusted for Age, Sex, and Ethnicity

**Model II:** adjusted for Age, Sex, Ethnicity, BMI, Diagnosis of KOA, History of hypertension, History of diabetes, History of coronary heart disease, Using PSI, ASA grade, Preoperative anemia, Use of tranexamic acid during surgery, and HOCA
